# Fewer Suicides: Due to Less Drinking and Increased Employment

**Published:** 1918-05-25

**Authors:** 


### FEWER SUICIDES.

### Due to Less Drinking and Increased Employment.

The war has had a remarkable and most unexpected effect in diminishing mental disorders of various kinds in the civil population. The Board of Control in England, and the Lunacy Board in Ireland, have both reported a diminution, the first recorded for fifty years, in the number of lunatics under the official cognisance of these bodies respectively, and now the Registrar-General reports a diminution in the number of suicides, and especially in the suicides of males. The rate per million of suicides by males in the period 1901-1910 inclusive was 157. In 1914 it was 151. In 1915 it fell to 104, or a diminution of about 30 per cent., which is very far beyond any ordinary fluctuation. Indeed, it was noted by Buckle, in his "History of Civilisation," that few statistics are more constant than those of suicide, and he makes this constancy the theme of an eloquent passage. In 1916 the rate per million rose to 111, still a great reduction on the pre-war rate. Suicides by females are always fewer than those by men, so that there is less room for shrinkage; but there has been a shrinkage, though it is not as great as in the case of males. In the ten years ending 1910, the number of suicides per million women was forty-seven; in 1914 and 1915 it was forty-five; and in 1916 it fell to thirty-eight.

Of course, the obvious explanation of the fewer suicides in males in this country is that so many males have left the country to fight abroad; but, like most obvious explanations, this will not hold water, for when we turn to the age distribution we find that the diminution in the number of suicides in males is greatest at the age of forty-five to sixty-five, that is to say, an age at which very few males went abroad in 1915-16. No. The figures leave no doubt that the diminution in the number of suicides is not only apparent, but is a real diminution. What can be the explanation?

Suicides are divisible into two distinct groups—suicides of the sane, and suicides of the insane. The number of the insane has diminished, and this would account for some small diminution in the number of suicides, but the number of the insane has been only slightly reduced, so that this explanation would not go very far, were it not that suicide and attempts at suicide are exceptionally frequent among those insane persons whose madness is due to drink, and the opportunities of obtaining drink have been very greatly reduced. Drink is an even more fertile cause of unsuccessful than of successful attempts at suicide; and it is noteworthy that the number of attempts at suicide known to the police has diminished even more than the number of successful suicides. This goes some way to corroborate the likelihood that a large part of the reduction in the number of suicides is due to the great reduction in the amount of drinking. This would especially affect men between the ages of forty-five and sixty-five, and it is between these ages that the suicide rate shows the greatest reduction. The chief motives of suicide in the sane are loss of interest in life and pecuniary ruin, and terrible as the effects of the war have been in other respects, yet in both these respects it has been beneficial. It has provided a subject of intense and enthralling interest, over and above the ordinary interests of life, to every man and woman in the country; and it has also brought, especially to the manual worker, the clerk, and the lower grades of skilled workers, unexampled prosperity. Wages in all occupations have risen by leaps and bounds, and although the cost of living has risen at the same time, it has not risen in anything like the same proportion. When boys of sixteen can earn, as many are now earning, £3, and even £5 per week, it is manifest that the general rate of wages has risen out of all proportion to the rise in the cost of living. No one who is capable of doing remunerative work need want remunerative work in these days, and as pecuniary loss, ruin, and destitution are the most fertile motives of suicide among the sane, there is little reason for wonder that the suicide rate has very greatly declined, especially among males between the ages of forty-five and sixty-five. For it is at forty-five that the difficulty of obtaining employment has in times before the war become acute.

Upon the whole, it seems that the diminution of drinking and the increase of employment are together sufficient to account for a large proportion of the reduction in the suicide rate, though other factors have no doubt been operative. Something may be due to the provision of a subject of universal and enthralling interest, as has already been suggested; but up to the end of 1916 the country generally did not take the war as seriously as has since been unavoidable. The war was not then brought home to the people in the same way. For one thing, there were then no food restrictions. Loss of interest in life, owing to the death of some person in whom the interest was absorbed, such as a sick mother or child, has been in the past a frequent cause of suicide, and it is now easier, nay, it it almost compulsory, to substitute for this lost interest the very present interest of a new and absorbing employment. Some reduction in the suicide rate may be put down to this. But an even greater reduction may be put down to employment itself. Dr. Watts, of pious memory, an authority now well-nigh forgotten, assured us that Satan finds some mischief still for idle hands to do, and trite as the maxim is, it contains a core of valuable truth. Nothing conduces so much to health, both bodily and mental, nothing conduces so much to happiness, as congenial and abundant employment: nothing tends so much to the making of the *malade imaginaire* and to *tedium vitæ* as idleness; nothing is so powerful an agent in producing recovery from madness as regular, persistent, and interesting employment; and never was there a time when regular

**FEWER SUICIDES—(*continued*).**

and interesting employment was so easily within reach of all, or so generally adopted. These factors, taken together, would seem enough to account for the statistics of suicide to which the Registrar-General calls our attention.

Though these statistics are eminently satisfactory as showing the prolongation of many useful lives that, but for the war, would have been self-sacrificed, yet they contain a warning. During the war, as during previous wars, employment is abundant, and money is plentiful. The nation is living on its capital. It is borrowing at a high rate, and living on the borrowed money. Inevitably there must be a bad time coming. Great wars are always followed by industrial depression, by lack of employment, want, and distress; and this is the greatest of all wars. We do not wish to croak, but it is only ordinary prudence to look ahead and forecast what is coming. All the experience of history goes to show that when this war is over there will be a period of great distress; and then it is to be feared that the reduction in the suicide rate will not be maintained.

